# Complex genetic and epigenetic regulation deviates gene expression from a unifying global transcriptional program

**DOI:** 10.1371/journal.pcbi.1007353

**Published:** 2019-09-17

**Authors:** Mónica Chagoyen, Juan F. Poyatos

**Affiliations:** 1 Computational Systems Biology Group (CNB-CSIC), Madrid, Spain; 2 Logic of Genomic Systems Laboratory (CNB-CSIC), Madrid, Spain; 3 Center for Genomics and Systems Biology, Department of Biology, New York University, New York, United States of America; Ecole Normale Supérieure, FRANCE

## Abstract

Environmental or genetic perturbations lead to gene expression changes. While most analyses of these changes emphasize the presence of qualitative differences on just a few genes, we now know that changes are widespread. This large-scale variation has been linked to the exclusive influence of a global transcriptional program determined by the new physiological state of the cell. However, given the sophistication of eukaryotic regulation, we expect to have a complex architecture of specific control affecting this program. Here, we examine this architecture. Using data of *Saccharomyces cerevisiae* expression in different nutrient conditions, we first propose a five-sector genome partition, which integrates earlier models of resource allocation, as a framework to examine the deviations from the global control. In this scheme, we recognize invariant genes, whose regulation is dominated by physiology, specific genes, which substantially depart from it, and two additional classes that contain the frequently assumed growth-dependent genes. Whereas the invariant class shows a considerable absence of specific regulation, the rest is enriched by regulation at the level of transcription factors (TFs) and epigenetic modulators. We nevertheless find markedly different strategies in how these classes deviate. On the one hand, there are TFs that act in a unique way between partition constituents, and on the other, the action of chromatin modifiers is significantly diverse. The balance between regulatory strategies ultimately modulates the action of the general transcription machinery and therefore limits the possibility of establishing a unifying program of expression change at a genomic scale.

## Author summary

How can we understand expression changes observed as a result of environmental or genetic perturbations? This issue has been conventionally answered by examining small groups of genes whose expression becomes qualitatively altered after these perturbations. But this approach is too simplistic, as we now know that extensive variation is typically observed. To explain this variation, recent works proposed a model in which genome-wide changes are explained by the action of a general program of transcription. Our manuscript reasons that given the complexity of eukaryotic transcriptional control, a unifying program of regulation cannot be achievable. Instead, we propose within an integrated framework of resource allocation that a rich structure of deviations from it exists and that by characterizing these deviations we can fully appreciate large-scale expression change.

## Introduction

The limited availability of the components of the cell expression machinery, for example, free RNA polymerases, cofactors, ribosomes, etc., creates a resource allocation problem that affects their activities. This differential allocation eventually represents a global program of regulation [1][2][3][4], with some genes expressed at the cost of others. As the dosage of these components can be modulated by the growth rate at exponential phase, the influence of the global program has typically been studied by quantifying how the expression of genes varies with growth conditions.

**Fig 1:**
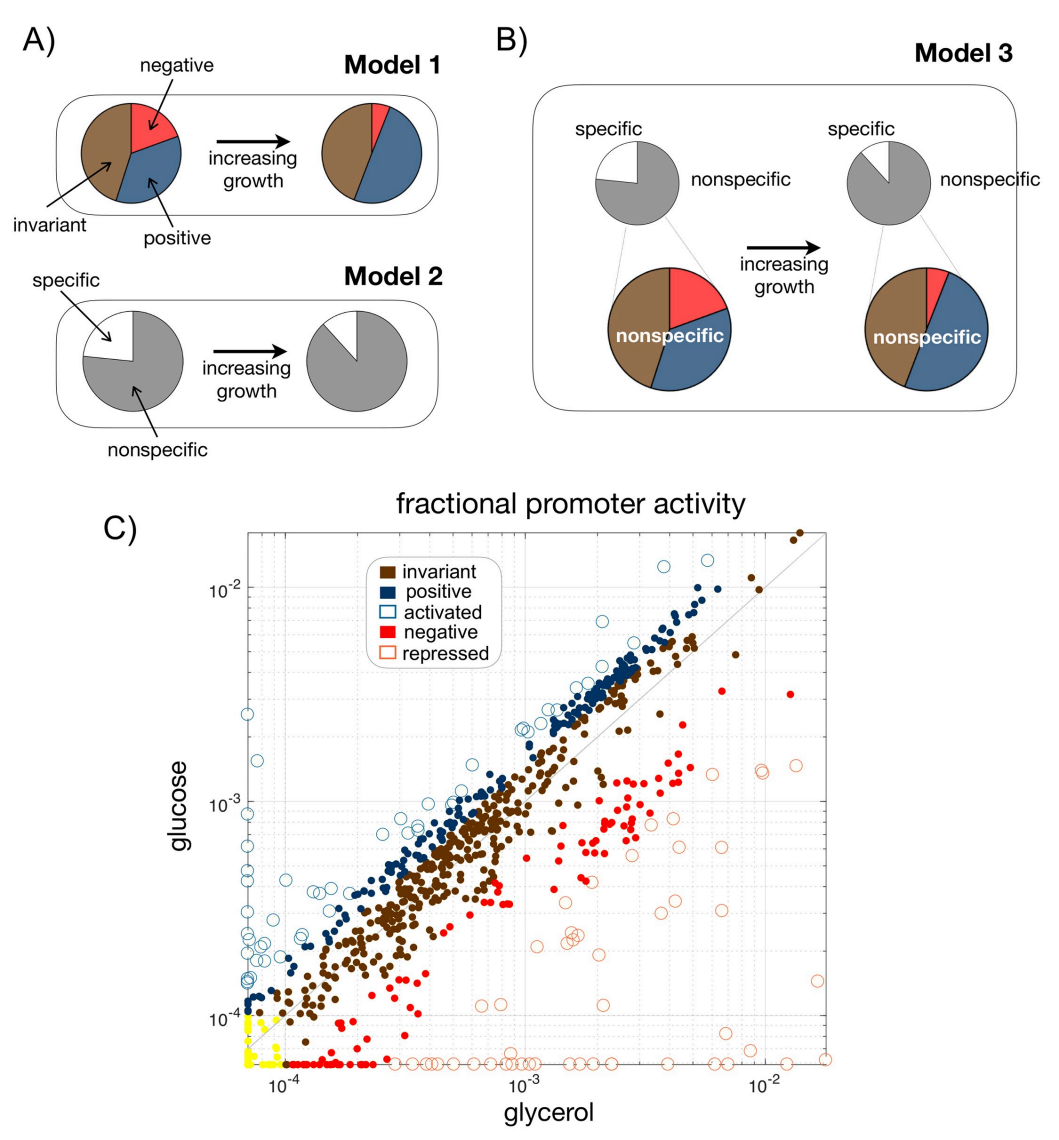
A new resource allocation model partitions the genome in five expression sectors. **A)** Top. Resource allocation model (Model 1) based on three sectors in which the expression of their constituent genes increases (positive genes, blue), decreases (negative genes, red), or remains constant (invariant genes, brown) with increasing growth rate. Bottom. Resource allocation model (Model 2) based on a specific and nonspecific sectors in which the expression of their constituent genes increases (nonspecific, white) or decreases (specific, grey) with increasing growth rate. **B)** Partition of genome expression into five sectors that combine the two previous models. A model-1-like partition appears as a fine structure within the nonspecific sector. We labelled this scheme as Model 3. **C)** Fractional promoter activity (fPA) for a transition between two example conditions increasing growth (glycerol to glucose). Promoters can be classified into the five sectors of model 3 depending on how their fPA changes (yellow dots indicate those with very low activity in both conditions). Repressed and activated promoters constitute the specific sector. Nonspecific promoters are constituted by one invariant type and two other subclasses whose fPA depends on the growing condition.

This led to the identification of laws that predict the expression of different cellular elements [3][4][5]. Two broad models have been considered. In a first model (model 1, Fig 1A, top), the impact of the global program is recognized by partitioning the genome in three minimal sectors [4][5]. One of them contains ribosomal genes whose expression increases with growth rate verifying its role in driving cell growth [6]. Two other sectors comprise genes whose expression decreases or remains invariant with growth. The three-sector model describes therefore fundamental aspects of cellular economics [7] already advanced in the early work of bacterial physiologists [8], and acts as a basic constituent to investigate many subjects. For instance, modifications to this framework were advanced to explain the cost of unnecessary gene expression [4], the dependence of cellular composition with antibiotics [9], or the rationale behind some seemingly wasteful carbon utilization strategies [10].

In a second model, the emphasis is not so much in the trade-offs between ribosomal genes and the rest, but between genes that follow a common pattern of expression and a minimal subset that diverges (model 2 containing two sectors: nonspecific and specific, Fig 1A, bottom) [11]. Here, the common pattern of expression is explained by a single scaling factor, which incorporates the resources of the global program that are not involved in the activation of the specific genes. This implies that most changes in expression result from a passive rather than active regulation what produces a unifying pattern that “simplifies” the expected complexities of genome-wide expression changes.

In this manuscript, we integrate both models (model 3, Fig 1B). The new framework discriminates a fine-structure within the nonspecific sector of model 2 that corresponds to the three-sector partition representative of model 1. While the integration of both views is significant *per se*–models 1 and 2 might appear unrelated–we also show that this scheme is fundamental to help us expose how specific regulatory strategies modulate the impact of the global transcriptional program at the genome-wide level.

To this aim, we first reexamined the original data that led to model 2, i.e., promoter activity (PA) measurements of *Saccharomyces cerevisiae*’s genes obtained in different growing conditions [11], to present our new scheme. However, this data only includes a subgroup of ~900 yeast genes, and thus the quantification of the resource allocation can only be approximated. To obtain a more precise classification we considered genome-wide expression data of yeasts growing in chemostats [12] and developed a method to define the new model at a large scale. We validate our partition through functional analysis of the corresponding components.

Armed with this new framework, we focused on understanding how the global program becomes modulated by specific regulatory mechanisms, a question which is only beginning to be addressed. Indeed, within the framework of model 1 results are mixed. For instance, several recent reports in bacterial systems demonstrated the prevalence of the global expression program, while they have lowered the importance of transcription factors (TFs) controlling the assumed deviations from it. TFs seem only to fine-tune the action of the global regulation [13][14] in combination with a few metabolites [15]. More recent work in yeast argued, in contrast, about the relevance of epigenetic factors (promoter nucleosomal stability) as modulators of the global program [16].

The function of the specific regulation might appear more straightforward *a priori* in the scheme of model 2. Specific genes are indeed enriched by specific regulation, generally coupled to the particular growth condition, whereas the behavior of those genes within the nonspecific sector does not seem connected to a particular transcription regulation strategy [11]. We thus examine within our new framework to what extent we could observe differential genetic and epigenetic regulation acting on the genes constituting each sector. We discovered well-defined regulatory patterns.

More broadly, our results put forward an integrated view of the resource allocation connected to genome-wide expression and emphasize how the global program is eventually modified by the specific regulation. Active strategies of control are certainly at work in genes within the fine-structure of the “nonspecific” response and can eventually associate the metabolic status of the cell with gene expression. Overall, this complex hierarchy of regulation in eukaryotes enhances the adjustment of genome-wide expression patterns beyond the one that could be achieved through a passive unifying program of global transcriptional control.

## Results

### Integration of two preceding resource allocation models

We first examined how PA changes with the growth rate and growth conditions for a subset of *Saccharomyces cerevisiae* genes. Keren *et al*. [11] presented a binary partition to describe these changes, recognizing a common response in the absolute PA values of most genes and a specific one in a much smaller subgroup. To focus on resource reallocation, we studied here fractional PA activities instead of absolute values, i.e., the fraction of PA of each gene in a given growth condition out of the summed activity of all genes in the set [3], and quantified their change for each pair of conditions (from low to high growth rate).

Fig 1C explicitly shows one of these transitions (glycerol to glucose conditions) to illustrate our general framework. The most extreme deviations of the global program correspond to the specific genes [11]. Note also that the stronger allocation of “expression resources” to these genes the fewer resources to biosynthesis (reduction of the nonspecific sector) affecting growth rate (Fig 1A, bottom). We revised the two-sector partition by further separating specific genes as specifically activated genes (fractional PA becomes much larger between conditions), and specifically repressed genes (fractional PA becomes much smaller), and also delimiting three components within the nonspecific sector: genes whose fractional PA remains approximately invariant (diagonal in Fig 1C), positive genes, whose fractional PA moderately increases between conditions, and negative genes (fractional PA decreases to only a limited extent, see Methods for details). These three sectors relate model 2 to model 1.

We validated this new partition in two ways. First, that a set of genes follows a precise proportional response between conditions was suggested by [11] as an evidence that they share a common functionality (e.g., being part of the same pathway, etc.). Here, we find that invariant, positive and negative genes also follow a precise proportional response (S1 Fig) suggesting that our analysis identifies a biologically relevant fine-grained structure. Second, we similarly expect that this fine-grained structure would parallel that proposed in model 1 [4] in terms of functional categories. This is the case. The invariant class is enriched by transcription regulation and ribosomal proteins; the latter being more extensively observed in the positive class. Indeed, positive genes are enriched by ribosomal genes (~65% of genes code for small or large subunits of the ribosome), while negative genes are enriched in ATP metabolic processes, e.g., oxidative phosphorylation or the TCA cycle (S1 Table, Methods).

### The five-sector resource allocation model on a genomic scale

**Fig 2:**
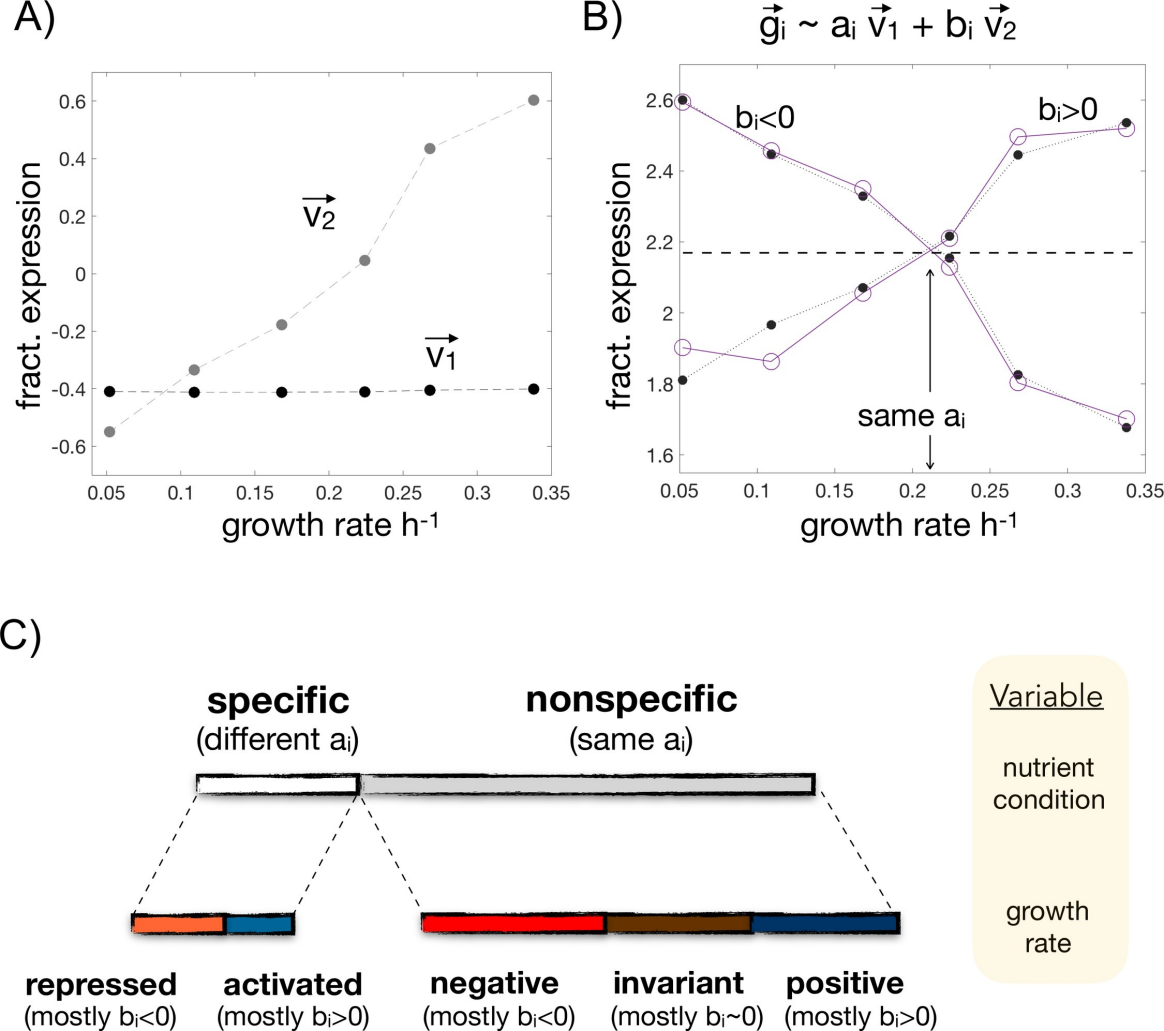
Genome-wide partition of gene expression in five sectors based on SVD. **A)** SVD components ${\vec{v}}_{1}$ and ${\vec{v}}_{2}$ describe baseline fractional expression and dependence with growth, respectively, and together explain most of the expression variance. **B)** The fractional expression of every gene ${\vec{g}}_{i}$ as a function of growth rate can be approximated by a linear combination of these two components, with loadings a_i_ and b_i_. We show two examples (purple circles denote the expression vector, while the black dots correspond to the two-component approximation; lines added to help visualization) with the same baseline (dashed line; same a_i_) but whose expression increases (b_i_>0) or decreases (b_i_<0) with growth. Data in A) and B) corresponds to growth in limiting glucose. **C)** The baseline expression of a gene can change, or not, between two nutrient conditions. A gene is nonspecific if its baseline expression remains the same (similar a_i_ loadings) in at least half of the 15 possible pairwise nutrient comparisons, being specific otherwise. Within nonspecific genes the second loadings b_i_‘s on each nutrient condition enable us to determine if the gene is positive (mostly b_i_>0 in all six conditions), invariant (b_i_~0) or negative (b_i_<0) with respect to growth rate change. Specific genes can similarly be separated between repressed or activated. In this way, we can partition the genome into five sectors what generalizes the scheme discussed with PA data in a subset of yeast genes (Fig 1). See main text for more details.

To substantiate the previous five-sector model based on ~900 genes, we examined a genome-wide DNA microarray dataset of yeast cells exhibiting the same range of growth rates for six different growth conditions defined by the limiting nutrient [12]. We studied again changes of relative expression (Methods) and applied singular value decomposition (SVD) to each nutrient separately. The first and second SVD components (Fig 2A) explain >90% of the variance in each condition (the components exhibited an analogous trend in all nutrients, S2 Fig). As a result, the fractional expression response to growth rate of each gene can be approximated by the linear combination of these two components (Fig 2B).

Furthermore, we can interpret the first element of the linear combination (${\vec{v}}_{1}$) as the baseline fractional expression of the gene, which does not change with growth rate, and the second element (${\vec{v}}_{2}$) as its monotonic response to growth (Fig 2B). This reading enables us to generalize the previous partition framework obtained with PA data. More specifically, a gene that maintains the same baseline expression (similar loading of ${\vec{v}}_{1}$ that we denoted a_i_) between two nutrients involves a nonspecific response. When this type of response in observed in at least half of all possible pairwise changes (>8) then we reason that the gene is nonspecific (recall that there are 6 different nutrients and consequently a total of 15 pairwise nutrient changes). Genes are considered specific otherwise (Fig 2C).

Beyond the classification above, the second component (${\vec{v}}_{2}$) provides a quantitative score (the second loading, b_i_) to classify genes as invariant, positive or negative (b_i_~0, b_i_>0, b_i_<0, respectively; Fig 2C. Methods). We labelled as invariant those nonspecific genes which exhibit this behavior in at least half of the nutrient conditions (>3 of a total of 6). Nonspecific and *not* invariant genes appearing more times as positive than as negative are categorized as positive, and likewise for negative. Finally, specific genes which appear more times as positive than as negative (again in all 6 conditions) are categorized as activated, and analogously for repressed (Fig 2C).

Finally, the functional analysis of genes within each sector agrees with that obtained with the PA data and previous reports, what substantiates the biological significance of the partition (S2 Table; see also S1 Table showing how this classification maps onto the subset of genes with PA data). In this way, we have generalized at a large scale the new resource allocation model, a genome-wide classification that we use throughout the next sections.

### TFs are usually nonspecific genes

We inspect next the influence of the most direct elements related to specific regulation, i.e., TFs. But before examining their impact on the activity of the global transcription program, we asked how TFs themselves are framed in the previous partition.

We observed that most TFs constituting the transcriptional regulatory network (122 of a total of 133 comprising the network, Methods) are nonspecific genes, i.e., they normally present similar basal fractional expression (a_i_ loadings) across all pairwise condition changes. Within this set, 31% exhibits an invariant response in more than half of the conditions (b_i_~0 in >3 nutrient conditions, of a total of 6), with five genes acting as invariant in all six conditions (*rsc1*, *mbp1*, *pho2*, *rgr1*, and *swi6*). Two of these (*mbp1*, *swi6*) are at the top of the network hierarchy (being involved in the mitotic cell cycle), and two are elements of relevant complexes that interact with RNA Polymerase II (*rsc1* of the RSC chromatin complex, and *rgr1*/*med14* of the mediator complex); they can be considered as elements of a general transcriptional machinery, for which maintaining the concentration invariant across conditions could be essential. Moreover, 32% of nonspecific TFs are mostly negative in all nutrient conditions, and only 4% mostly positive. Of note, some of the negative TFs–whose expression decreases with growth (b_i_<0)–are positive regulators of transcription in response to stress (e.g., *bur6*, *gcn4*, *rpn4*), which justifies their overexpression at low growth rates.

### Genes within each partition sector show different degrees of transcriptional regulation

**Fig 3:**
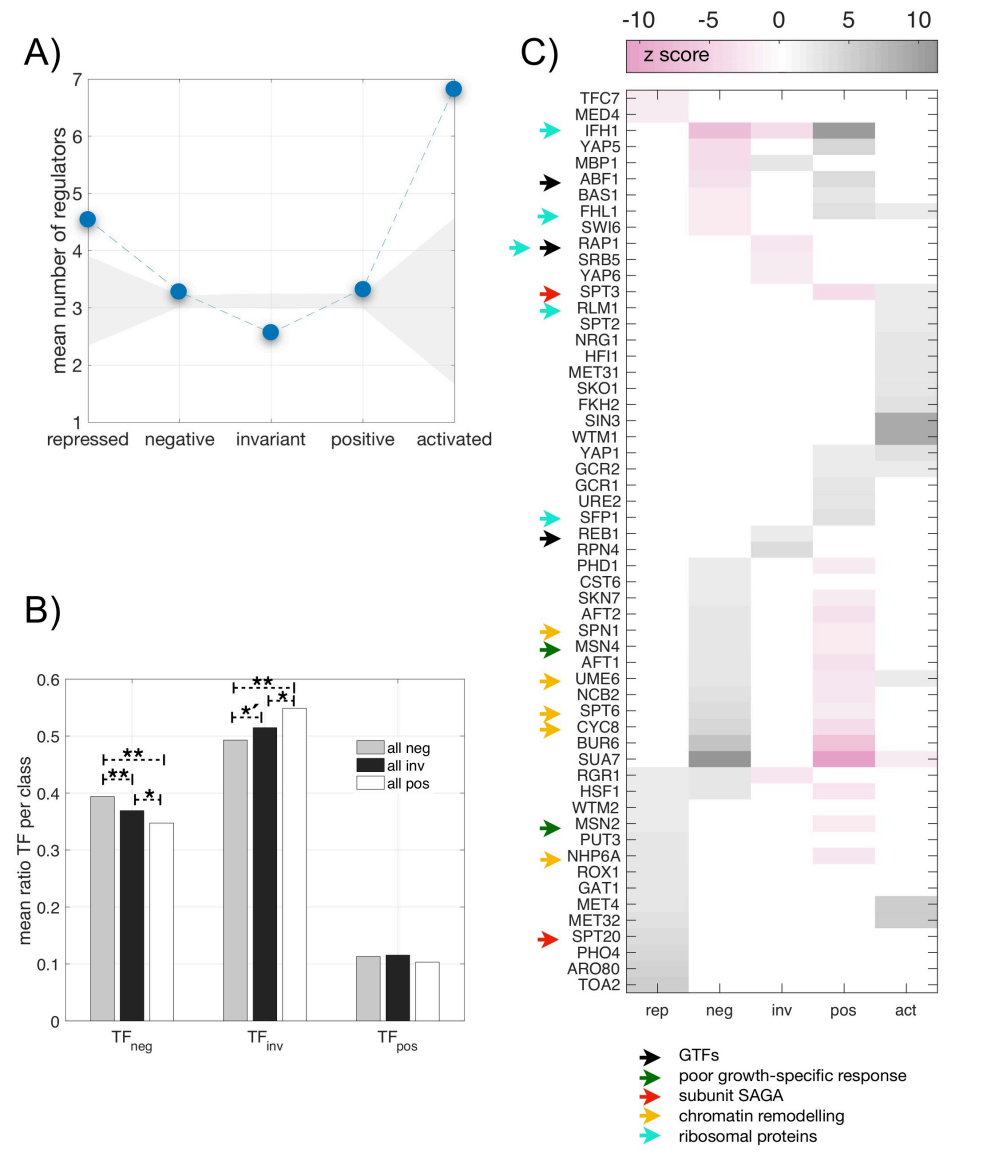
Differential integration of transcriptional regulation and the five-sector resource allocation model. **A)** Mean number of regulators acting on genes as a function of their response to growth (blue dots; dashed line to help visualization). Grey shading denotes the average null values +/- 2 standard deviations obtained by randomization. **B)** Mean ratio of the fraction of TFs of a given class with respect to growth (e.g., TF_neg_ denotes TFs which are negative genes) for each group of target genes (also for a given class; here we do not distinguish between nonspecific and specific). Histogram obtained in glucose conditions, see also S3 Fig for other nutrients (** p < 0.001, *´ p < 0.01, * p < 0.05, two-sided KS test). **C)** Regulators that act dominantly, or secondarily, in genes showing a significant regulatory coherence. Color denotes z-score with respect to a null obtained by randomization (positive values denoting enrichment; negative values denoting lack of it). Properties of some regulators are also included (arrows). See main text for details.

Is the extent of regulation of target genes dependent on which sector they belong to? We computed the mean number of regulators acting on genes within each sector. Nonspecific genes are less regulated, on average, by TFs than specific ones as expected [by 3.09 TFs *vs*. 5.06 TFs, p = 1.20 10^−4^, two-sample Kolmogorov-Smirnov (KS) test]. Within nonspecific genes, invariant genes are less regulated than nonspecific and *not* invariant ones (by 2.56 TFs *vs*. 3.3 TFs, p = 8.16 10^−13^, two-sample KS test). Finally, nonspecific and positive genes are slightly more regulated than nonspecific and negative genes (by 3.33 TFs *vs*. 3. 27 TFs, p = 0.0018, two-sample KS test). Overall, specific genes are subjected to more regulation (larger number of TFs), while nonspecific and invariant ones show the least (Fig 3A).

### TFs and their regulated genes frequently exhibit the same response to growth

Although Fig 3A shows how the number of transcriptional interactions is reflected differentially in the sectors of the partition it does not assure us whether these interactions are functioning, e.g., regulation by TFs have been shown to play a minor role during physiological transitions in bacteria [13]. To evaluate this, we examined several features.

We initially inspected if target genes presenting a particular growth response are enriched by TFs showing the very same response, as the similarity of the responses could indicate that these TFs do influence the expression of the target. We quantified this similarity for each nutrient condition (glucose, ammonium, etc.) and considered again the loading of the second component to define TFs or target genes as presenting negative, invariant or positive responses to growth rate (b_i_~0, b_i_>0, b_i_<0, respectively, Methods). We thus computed–for each target gene–the fraction of its regulators that behave as negative, invariant, or positive with the growth rate in a given nutrient (TF_neg_, TF_inv_, TF_pos_, respectively). For the glucose condition, Fig 3B shows the mean of these fractions for target genes which themselves display a negative, invariant, or positive response, respectively.

TFs exhibiting a negative response are more likely to be found acting on target genes that are also negative (higher mean TF_neg_ on negative genes), while invariant (TF_inv_) and positive (TF_pos_) TFs regulate more often invariant and positive target genes, respectively (the latter signal is weaker and depends on the particular condition, S3 Fig). In sum, TFs that exhibit the same behavior as their cognate target gene with respect to growth tend to predominate, on average, on its regulation; this suggests that part of the regulatory structure is functional.

### Specific TFs regulate genes fitting to each partition sector

To further assess the active regulatory impact of TFs, we measured the correlation of the response to growth rate between any particular gene and all its cognate TFs (“regulatory coherence”, S4 Fig) and then calculated if this correlation is statistically significant (Methods). The fraction of specific genes whose regulatory coherence is larger than expected by chance is superior to that of nonspecific ones (S5A Fig), and the former also show significant coherence in a greater number of nutrient conditions (S5B Fig). Both results imply an existing contribution of TFs to deviate gene expression from the global program.

Can we identify those TFs which take part in the statistically significant coherent regulation? Fig 3C shows that within this set some TFs acts principally on genes belonging to a precise sector of the partition more than anticipated by chance (Methods). One can identify here that TFs coherently controlling specific genes are normally not acting on nonspecific ones [this is supported by earlier reports[17]]. Moreover, some of the TFs that work coherently on positive genes (gray color, Fig 3C) are particularly absent in negative ones (pink color, Fig 3C) and *vice versa*. In addition, those that coherently control nonspecific genes are higher up in the network hierarchy (Methods). We also noted that some of these TFs are involved in chromatin remodeling (Cyc8, Ume6, Spt6, Msn4, Abf1, Msn2, Nhp6A, acting on nonspecific ones; Hypergeometric distribution’s p-value = 1.5 10^−9^, and Holm-Bonferroni multiple testing correction), or chromatin organization (Spt3, Spt2, Pho4, FKh2, Sin3, Spt20, Wtm2, Wtm1, Hif1, acting on specific genes; p-value = 5.8 10^−8^, distribution and multiple testing as before). We examine epigenetic aspects next.

### The resource allocation model also reveals distinctive epigenetic regulation

**Fig 4:**
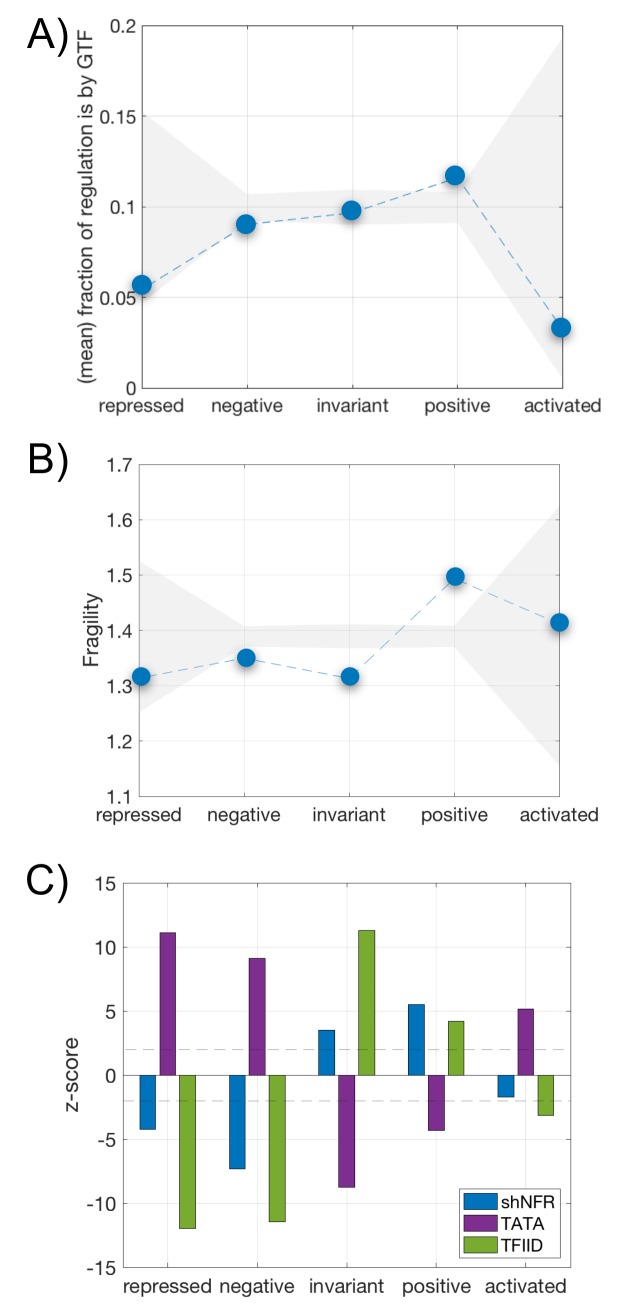
Differential integration of epigenetic regulation and the five-sector resource allocation model. **A)** Mean fraction of global transcriptional regulators (Rap1, Abf1, Reb1, Cbf1 and Mcm1) within the full set of regulators acting on each gene. Grey shading denotes the average null values +/- 2 standard deviations obtained by randomization. Dashed lines to help visualization. **B)** Mean nucleosomal fragility. Shading/lines as before. **C)** Enrichment of nucleosomal free regions (shNFR, blue), presence of TATA boxes (purple), or action of TFIID global factor (green) as function of response class (measured as z-score with respect to a null by randomization; dashed line indicates z-score = +/- 2).

To inspect the influence of the epigenetic control mechanisms, we first quantified the proportion of general transcription factors (GTFs) found within the set of TFs acting on a given gene [18]. GTFs (Rap1, Abf1, Reb1, Cbf1, and Mcm1) usually have little intrinsic regulatory activity and comprise–together with the presence of chromatin remodelers (in particular, RSC–Remodeling the Structure of Chromatin)–an alleged general machinery of expression. We observed that GTFs constitute a significant fraction of the TFs acting on of positive genes, while the opposite is observed for negative ones (Fig 4A).

GTFs are also associated with particularly fragile nucleosome promoter architectures [19], a connection recently investigated [16]. Using this data, we computed the nucleosome landscape for the different gene classes (Methods). Promoters of positive genes are certainly enriched in fragile nucleosomes (Fig 4B) while both negative and invariant genes lack these architectures. Positive genes are therefore more sensitive to adjust the global program of expression by means of chromatin modulation. Enrichment of other promoter features contribute to this observation (Fig 4C, Methods), like the absence of TATA boxes [20], the action of TFIID over SAGA [21] [but this precise grouping has been recently reexamined [22]], the presence of nucleosomal free regions closer to the transcriptional starting site (shNFRs) [23] (partially associated to the previous score of fragile nucleosomes), and the dominant effect of *trans* variability (S6 Fig) [24].

### Chromatin modifiers further modulate the global program

Finally, we examined the effects of mutating different types of *trans*-acting chromatin regulators on the genes constituting the sectors using a previously assembled compendium [25] (Methods). We considered first the magnitude of the change of expression (i.e., *absolute* values of expression) before and after the mutation of several types of modifiers. With the exception of histone acetyltransferases (HATs) and TATA-binding protein related factors (TAFs), the influence of most chromatin modifiers is stronger in specific genes as compared to nonspecific ones (S7 Fig, Methods), which implies that some TFs require the recruitment of chromatin modifiers to act [25].

**Fig 5:**
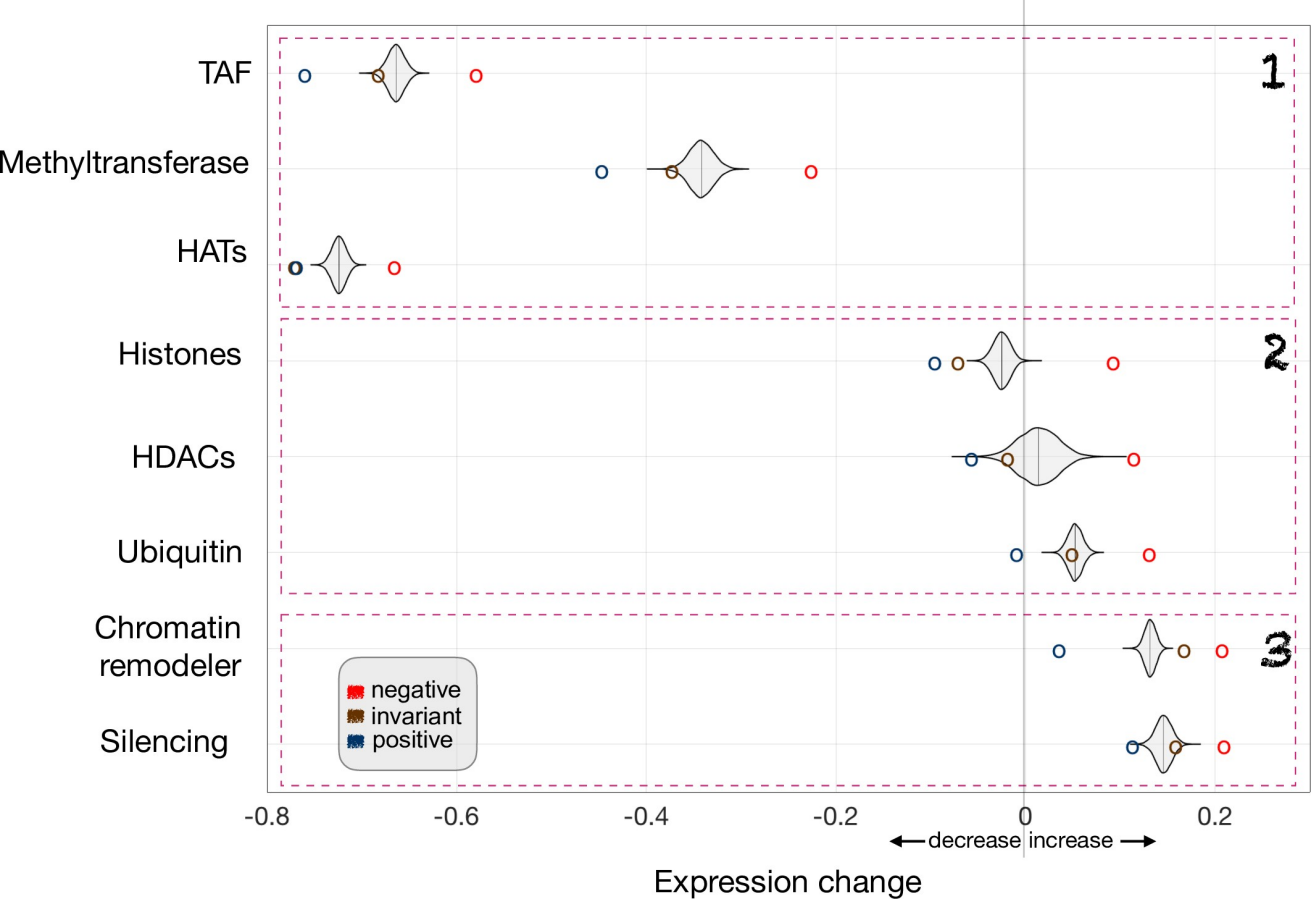
Chromatin modifiers act differentially on nonspecific genes. Mutations in chromatin modifiers reveal their diverse regulatory role. For each modifier, we plotted its mean effect, after mutation, on the genes constituting the negative (red circle), invariant (brown circle), or positive (blue circle) sectors. A kernel density plot corresponding to a null distribution obtained by permutation is also shown (Methods). We observed three broad categories: 1/ modifiers acting as activators (mutation decreases significantly the expression of invariant/positive genes), 2/ dual activator/repressor (mutation decreasing/increasing significantly expression of positive/invariant or negative genes, respectively), and 3/ repressors (mutation increasing significantly expression of negative genes). TAF: TATA-binding protein related factors; HATs: histone acetyltransferases; HDACs: histone deacetylases.

Within nonspecific genes, we also quantified the type of expression change experienced after mutation of the modifiers and found three broad relationships (Fig 5): 1) Epigenetic regulators acting as part of a general machinery (HATs–including SAGA–, TAFs and methyltransferases) whose mutation causes a general decrease in expression, very particularly in invariant and positive classes. Indeed, work by [22] and [26] demonstrated that SAGA and TFIID are recruited to RNA Polymerase II promoters genome-wide and that each complex is generally required for Polymerase II transcription, i.e., its mutation would lead to a genome-wide decrease of gene expression. 2) Regulators (e.g., histones, etc.) acting in a dual manner: increasing the expression of negative genes after mutation (remodeler as a repressor) or reducing their expression in positives (remodeler as an activator). This underlines the enrichment of negative and positive classes by stress and ribosomal genes, respectively, which are largely regulated in an opposite manner [27]; a dual role of remodelers as activators and repressors have been previously reported [28][29]. And 3) regulators as broad repressors (mutation increases expression), e.g., those that represent regulation by gene silencing.

## Discussion

Recent studies discussed how most genome-wide expression changes might be the consequence of a unifying global program of transcription control. This program is determined by the availability of the pooled components of the expression machinery and necessarily refers to a problem of distribution of these resources for the activation of distinct parts of the genome.

To quantify the impact of this program, resource allocation models typically frame then the problem in terms of genomic partitions. We follow here this approach to divide the yeast genome into five sectors that integrate two earlier models, not connected until now [6][11]. Essential to our framework is the ability to discriminate what it was contemplated before as a common nonspecific proportional response subjected to the same global regulation [11] into three subclasses: invariant genes, that best follow the global program, and positive and negative genes, which were broadly defined in other studies as growth-related genes [12][30][31]. The analysis of relative expression values is important, as it allows us to appreciate expression rearrangement among partition sectors [3][4].

With our new scheme, we first explore the impact of specific genetic regulation. How this regulation fine-tunes the global program is largely an open question with only a few preliminary studies in bacterial systems, which suggested a secondary role of TFs [13][14][15]. We consider yeast data on transcriptional regulation to first recognize invariant genes as the ones that are the least regulated by TFs. The degree of regulation and also the regulatory coherence increases among the rest of nonspecific classes, and between these and the specific ones. Note that coherence quantifies here the similarity of expression response to growth rate (for a given nutrient condition) between genes and the TFs acting on them. A strong signal suggests an operative role of the latter, which contributes to the departure of the expression of these genes (specific genes but also negative and positive ones) from that anticipated by the only effect of the global program.

Among those TFs whose action is particularly coherent, we detect groups that almost exclusively regulate specific or nonspecific genes, or positive and negative genes, i.e., the functioning of the TF network is somehow segregated. These results imply overall that TFs do play a role as coordinators of gene expression during physiological transitions in eukaryotes what partially opposes the dominance of the global program previously proposed in bacteria [13].

Beyond TF regulation, we discriminate two general promoter architectures. Those that are TATA enriched and shNFR/TFIID depleted (found in nonspecific negative and specific genes), and those that are TATA depleted and shNFR/TFIID enriched (presented by the nonspecific invariant and positive genes); features that are similarly observed in housekeeping an stress-related genes [20][21][22][23] (Methods) and metazoans promoters [32] (Type I promoters, genes expressed in a tissue-specific manner, and Type II promoters, ubiquitously expressed genes, respectively).

That (nonspecific) positive genes are moderately controlled by TFs (like negative genes) but depleted in TATA box (unlike negative) could suggest certain expression features (e.g., high level of transcription, S8 Fig) and alternative modes of epigenetic regulation. Indeed, positive genes are enriched in fragile nucleosomes, which highlights the regulatory role of nucleosomal stability [16]. This is supported by the particular action of GTFs on these genes, as GTFs modify nucleosomal stability [19].

Furthermore, epigenetic modifiers adjust gene expression of the nonspecific genes in a distinctive manner by three main configuration types: 1/ general activators of invariant and positive genes, 2/ remodelers working in a dual manner; repressors of negative genes and activators of positive ones, and 3/ elements acting as general repressors of negative genes. Note that the second configuration (type 2) might further strengthen the consequences of the passive control of nonspecific genes, and contributes overall to an intricate balance of genetic and epigenetic systems that, together with the global control program, coordinates eukaryotic genome-wide expression change.

### Global and specific regulation in a broader context

How can we then explain the monotonic variation of fractional expression of the genes in the negative and positive partition components if, as suggested by Hansen and O’Shea [33], some TFs can mostly transmit qualitative (presence/absence of a particular nutrient) rather than quantitative (amount of nutrient) information.

One explanation is that this monotonic variation is the result of cell population shifts with growth rate, instead of changes in single-cell resource allocations. Indeed, Brauer *et al*. (12) observed a decreasing fraction of unbudded cells (proportion of cells in G0/G1 division cycle) as populations grew faster. However, variations with growth rate cannot solely be attributed to the fact that slow-growing cells enlarged their G1 cell cycle phase as neither [12] nor we observed a bias in positive/negative genes with any particular phase of cell cycled genes.

Another explanation is that the variation could also be part of an intrinsic and mechanistically complex environmental stress response (ESR) [34], so it is interesting to examine how the genes linked to this response fits into our partition. These genes included two complementary groups representing rather small subsets within the partition sectors: induced ESR genes enriched in nonspecific negative and repressed genes and repressed ESR genes enriched in nonspecific positive genes (Methods). Thus, ESR genes appear to represent a particularly extreme case of deviations to the global transcriptional program. While previous studies indicated that part of these genes might still be associated to cell cycle cell populations shifts [35], Ho *et al*. [36] recently discussed the preservation of a core ESR signal after controlling for these effects.

But maybe the dominant explanation is metabolism, which is highly sensitive to the limiting nutrient [37] and can act as a regulator of many of the epigenetic factors discussed above. Indeed, several metabolites (e.g., GlcNAc, NAD^+^, acetyl-CoA, alpha KG, ATP) are known to regulate transcription through interactions with enzymes involved in epigenetic modifications [38]. For example, acetyl-CoA induces cell growth and proliferation by promoting the acetylation of histones at growth genes [39] (histone acetylation affects rather similarly specific and nonspecific genes, S7 Fig, which supports its potential role as a widespread mechanism).

All these previous readings contribute to a general picture in which it can be conceived two ways to coordinate gene expression to the available nutrients: a regulation by signaling pathways, i.e., specific responses, that dictates growth rate by sensing a certain environmental condition (feed-forward), or a mechanism that senses growth rate, or another related internal cell variable, and then modifies expression (feedback)[40]. In this context, Model 2 (Fig 1A, bottom) could explain that the global program is responding always to the environment (feed-forward), although indirectly since it can only use those resources that were not consumed in the mounting of the specific response. This agrees with the observation that ribosomal genes (representatives of the nonspecific program in Model 2) follow the feed-forward path [41]. The fine-grained structure of the nonspecific class (invariant/positive/negative), Model1 (Fig 1A, top) could nevertheless monitor growth rate, at least partially, with the feedback being mediated by epigenetic mechanisms driven by the metabolic state of the cell (see paragraph above).

In this work we have studied changes in fractional expression but not in mRNA abundances. It is known that the global program dictates that the faster a population of cells growths, the higher the promoter activity (rate of RNA synthesis) [11] or total mRNA abundance (rate of RNA synthesis and degradation) [42]. We expect most (if not all) gene products to follow this (absolute) global program, with potential additional layers of regulation (which are nutrient and gene dependent) that increases or decreases mRNA levels. Expression of the genes within the invariant group best follows the absolute global program, with positive genes being slightly above -and negative genes slightly below- this response (but all of them incrementing mRNA levels or promoter activities) (e.g., S1B Fig). On the other hand, it would be interesting to quantify the degree to which single cells can present a distribution of resources that is separated from the model here discussed [43], as well as to understand the mechanisms that lead to such divergence.

In summary, although one could argue that cellular physiology can indeed determine a global transcriptional program of gene expression control, our work highlights that this program is adjusted by the integration of effective genetic and epigenetic modes of regulation. This modulation limits the prospect of “simplifying” our understanding of genome-wide expression change and calls for a combination of mechanistic and phenomenological approaches–like the work presented here–to finally unravel such complexity.

## Materials and methods

### Promoter activity (PA) data

Keren *et al*. [11] measured the activities of ~900 *S*. *cerevisiae* promoters in 10 different growing conditions using a library of fluorescent reporters. For each strain in every growth condition, PA was obtained as the YFP production rate per OD per second in the window of maximal growth.

### Five-sector partition based on PA data

We computed fractional PA (f_PA_) for each growth condition as the ratio of the PA of each gene to the summed PA of all promoters (for a gene *i*, f_PA,i_ = PA_i_ / Σ_i_ PA_i_). Ratios of f_PA_s for each pair of conditions (with increasing growth rate) were also calculated. We then computed the absolute distance of these ratios to ratio 1 (i.e., same f_PA_ in both conditions), and defined as invariant genes the top 350 genes (distance closest to 0) and as activated (repressed) the bottom 50 with ratio >0 (< 0). The rest of genes with ratio >0 (<0), and both f_PA_s > 10^−4^, were designated as nonspecific positive (negative). We used the “typical” class of a gene (the most frequently occurring category that a gene presents in all pairwise growth rate changes) to functionally characterize the sectors (S1 Table) what confirmed their biological significance. Minor modifications on the threshold values defining these sectors did not alter the conclusions.

### Microarray data

Brauer *et al*. [12] grew yeast cultures in chemostats under different continuous culture conditions (six different limiting nutrients each at six dilution rates) and measured mRNA abundance with two-color microarrays. Since the original reference channel for all samples corresponded to a particular glucose condition, which mixes the response of different nutrients, we reanalyzed the data without this reference by considering the red processed signal as independent channel [44], and normalizing by the corresponding sum for each case to obtain a fractional score; more specifically, the fractional expression value of gene *i* is given by f_i_ = log_10_[10^6^(g_i_/Σ_i_ g_i_)], with g_i_ being the corresponding red-processed signal. SVDs were computed on this processed data.

### Five-sector partition based on microarray data

We defined as nonspecific genes those whose difference on the loadings of the 1^st^ component (a_i_’s) between two conditions is less, or equal, than three standard deviations of all gene differences (in absolute values). Genes are otherwise considered specific (activated or repressed if the difference of a_i_’s is positive or negative, respectively). Moreover, absolute values of the loadings of the 2^nd^ component (b_i_’s) were sorted to define those with smallest values (top 2500) as invariant genes, with the rest being positive or negative (determined by the sign of b_i_). To define the partition, we classify as nonspecific those genes that act as nonspecific in >8 pairwise conditions (out of 15). Nonspecific genes acting as invariant in > 3 conditions (recall that the total number is 6) are labeled as invariant. Nonspecific and not invariant genes appearing more times as positive than as negative (in all 6 conditions) are categorized as positive, and likewise for negative. Specific genes which appear more times as positive than as negative (again in all 6 conditions) are categorized as activated, and analogously for repressed. The number of genes for each category is then (repressed, negative, invariant, positive, activated) = (70, 2503, 1749, 1914, 20). This partition is the one used for all the regulatory analysis (S2 Table shows the connection between these sectors and those obtained with PA data) with the exception of Figs 3B and S3 in which only the second loading was considered to classify all genes as positive, negative and invariant. Finally, note that minor modifications on the threshold values defining these sectors did not alter the conclusions and that the biological significance of the sectors is eventually validated by the regulatory and functional signals observed throughout the results.

### Regulatory network

Regulatory data was obtained from http://yeastmine.yeastgenome.org. We used data from different manuscripts using chromatin immunoprecipitation, chromatin immunoprecipitation-chip, chromatin immunoprecipitation-seq, combinatorial evidence, and computational combinatorial evidence, but discarded microarray data to avoid any possible circularity. The network consists of a total of 20,673 interactions with 133 TFs involved whose expression was also quantified in Brauer *et al*. [12]. We also computed the hierarchical organization of the network using a vertex-sort algorithm, which first finds the strongly connected components of the network to then apply an iterative leaf removal algorithm [45]. The network has three hierarchical levels. Bas1, Mbp1, Med6, Spt7, and Swi6 appear at the top of the hierarchy.

### Regulatory coherence

We identified the set of TFs regulating each gene and quantified the Pearson’s correlation coefficient between the expression vectors (as a function of growth rate) of each TF within the set and the target gene, to then take the mean (S4 Fig). This quantity is what we termed regulatory coherence, which is computed for each particular nutrient condition, i.e., six scores corresponding to the six different nutrients. The resulting scores are compared to a null distribution to assess their statistical significance (we computed a z score). This distribution is obtained by randomizing the expression vectors for each gene (again for a given nutrient), 1000 times, and then calculate the equivalent regulatory coherence. We define those genes with the z score > 2 as the ones displaying significant regulatory coherence. Finally, to identify those TFs that are acting more significantly on each partition component we first measure the proportion in which each TF acts on (significantly) coherent genes, within the five-sector partition, and then estimate the extent that this proportion departs from a chance expectation by randomization of the partition classes. This enables us to compute the z score shown in Fig 3C.

### Fragile nucleosome data

Nucleosome occupancy and position have been measured by analysis of MNase-digested chromatin. Recent work noted that certain nucleosomes were extremely sensitive to this digestion, and thus obtained a quantitative score of nucleosome fragility that we used for our analysis, S6 Table in [16].

### Promoter properties

We showed in the main text that positive genes are enriched in promoters displaying fragile nucleosome architectures, which have been discussed recently as an epigenetic mechanism of regulation. Other features contributed to the sensitivity to regulation of this class, like the absence of TATA boxes, the action of TFIID over SAGA, etc. Our results build on previous studies that showed how different genes exhibit different control strategies to regulate expression [20,21,23,27]. For instance, it was observed that housekeeping (constitutive) genes where enriched among those with TATA-less promoters and related to TFIID transcription, in contrast to stress-related genes enriched in among TATA-box promoters, and regulated by SAGA complex. This enrichment of promoter features further validates the biological significance of the partition.

### Chromatin compendium

This set includes 170 gene expression profiles for chromatin-regulation related mutations (expressed in log_2_ ratios) taken from 26 different publications [25]. It covers more than 60 potential interacting chromatin modifiers such as histone acetyltransferases (HATs; the NuA4, HAT1 and SAGA complexes), histone deacetylases (HDACs; the RPD3, HDA1 and SET3 complexes), histone methyltransferases (the COMPASS complex), ATP-dependent chromatin remodelers (the SWI/SNF, SWR1, INO80, ISWI and RSC complexes), and other chromatin-affecting genes and cofactors such as Spt10, Sir proteins and the TATA-binding protein (TBP). We normalized each dataset to unit variance [24]. For S7 Fig, we took absolute values to estimate the strength of the chromatin regulator effect. Note here that growth rate reduction can be connected to the impact associated to many of these deletions, so we controlled for the possible contribution of cell cycle population shifts as described (next section). This enables us to better identify expression changes due to regulation [35].

### Removal of the slow growth signature

We took the full data in [46] to obtain the slow growth profile and remove the slow growth signature in the epigenetic data following [35]. In brief, the slow growth profile is obtained as the first-mode approximation of the data after SVD decomposition. To compare with the epigenetic compendium data, we chose the column of this approximation with the largest norm as the slow growth signature. The correlation with the slow growth signature is removed by transforming the epigenetic data in Gram-Schmidt fashion by subtracting from their projection onto the basis vector, given by the normalized slow growth profile.

### Statistical analysis

Null models associated to most results are obtained by randomly assigning each gene to one of the sectors, to then compute the precise statistic (e.g., mean number or regulators in Fig 3A). We typically considered 10000 randomizations unless it is stated otherwise and show the mean and +/- 2 std deviations of the corresponding statistic (e.g., gray shading in Fig 3A). Moreover, all p values shown in enrichment analyses (S1–S2 Tables) are calculated using the Hypergeometric distribution with Holm-Bonferroni correction for multiple testing.

### ESR genes

There are 281 stress-induced and 585 stress-repressed genes–as defined in [34]–within the set of genes delineating the five-sector partition. A subset of nonspecific negative genes and specific repressed genes corresponds to stress-induced (232 out of 2053, and 10 out of 70, respectively), while a subset of nonspecific positive genes corresponds to stress-repressed (485 out of 1914). Note that most of the features discussed in the main text associated with the five-sector partition remain when controlling for ESR genes.

## Supporting information

S1 Fig Scaling factors between pairs of conditions. **A)** Genes that follow a single proportional scaling may serve a definite cellular function according to [11]. We find a single scaling that describes the change of promoter activity (PA) for different subsets of promoters according to the five-sector partition. The three classes within the nonspecific promoters (invariant, negative, positive) clearly show singular scaling. Shown also a null that corresponds to the ratio of growth rates between conditions (cyan curve)**. B)** PA response of a typical invariant, positive and negative gene that corresponds to the *mrs11*, *rps6A* and *atp5*, respectively (conditions sorted by increasing growth rate; this is absolute PA not fractional PA). A null model of the dependence of PA with growth rate is given by the ratio of growth rates (empty circles). Gene categories within the global group clearly separate from the null.
(TIF)

S2 Fig SVD components for all nutrients. First and second SVD components exhibited an analogous trend in all conditions what underlines a core response. As a result, expression of each gene can be approximated by the linear combination of these two components on each nutrient.
(TIF)

S3 Fig Association between class of TF and class of cognate target gene for all nutrients. Fraction of TF class (negative/invariant/positive) acting on target genes divided also with respect to growth response (negative/invariant/positive; nonspecific and specific genes were included that we denoted as allneg, etc.). Mean values of each grouping are shown in bars, while the orange curves show the distribution of each class of TF on each condition.
(TIF)

S4 Fig Regulatory coherence. To estimate the active regulatory character of TFs, we measured the Pearson’s correlation of the response to growth rate between a particular target gene and all its cognate *n* TFs to then take the mean. This is the (mean) regulatory coherence in a given nutrient condition.
(TIF)

S5 Fig Regulatory coherence and the five-sector resource allocation model. **A)** Percentage of genes within each class whose regulation is significantly coherent in at least one nutrient condition. Note that invariant genes show minimal coherence. **B)** Percentage of genes within each class that exhibits significant regulatory coherence in 1 to 6 different nutrient conditions. Specific genes (both repressed and activated) exhibit more cases of genes significantly coherent in more different conditions, while invariant genes show the opposite. See Methods, main text, for details.
(TIF)

S6 Fig *Cis* and *trans* variability with respect to the five-sector resource allocation model. A cross between a standard laboratory yeast strain and a wild isolate allowed the computation of *cis* and *trans* effects on transcriptional variance [24]. For each partition, we quantified the mean of these measures and showed the associated z-score with respect to a null by randomization; dashed line indicates z-score = +/- 2. Positive genes show dominant effects associated with *trans* variability (trv and civ denote *trans* and *cis* variability, respectively).
(TIF)

S7 Fig Chromatin modifiers act differentially on specific and nonspecific genes. The chromatin regulatory effect (CRE; *x* axis) quantifies change in gene expression (absolute value) due to mutations in chromatin modifiers. CRE values larger than expected by a null (z-scores > 2, obtained by randomization) are observed for most modifiers on specific genes. Each circle type corresponds to a class of epigenetic modifier (TAF: TATA-binding protein related factors; HATs: histone acetyltransferases; HDACs: histone deacetylases); *y* axis denotes z-scores and dashed lines emphasizes z-scores within +/- 2 values.
(TIF)

S8 Fig Expression abundance and noise with respect to the five-sector resource allocation model. Mean expression and protein abundance (A) and protein noise (B) with respect to the five-sector partition as compared to a null in which classes were assigned randomly (10000 randomizations; y-axis is plotting the associated z-score, shading corresponds to z-score values within a range of -/+ 2; SD/YEPD denote poor/rich growing conditions). Nonspecific and positive genes showed high expression and low noise, a signal that was associated to the presence of fragile nucleosomes in the promoter and the action of general transcription factors [both enriched in nonspecific positive genes, see main text and [27] for details on data].
(TIF)

S1 Table Five component partition from the promoter activity dataset by Keren *et al*. [11]. This includes: 1/list of genes included in each of the five components, and their correspondence to the genome-wide partition (see S2 Table), and 2/functional enrichment analysis of each component (GO process, Kegg Pathways and UP_KEYWORDS).
(XLS)

S2 Table Genome-wide five component partition, from the gene expression dataset by Brauer *et al*. (12). This includes: 1/list of genes included in the five components, and 2/functional enrichment analysis of each component (GO process, GO cellular component, GO molecular function).
(XLS)
