# Supplementary figures and images for: Complex genetic and epigenetic regulation deviates gene expression from a unifying global transcriptional program
Source: PLoS Comput Biol. 2019 Sep 17;15(9):e1007353. doi: 10.1371/journal.pcbi.1007353 (PMC6764696; doi:10.1371/journal.pcbi.1007353)

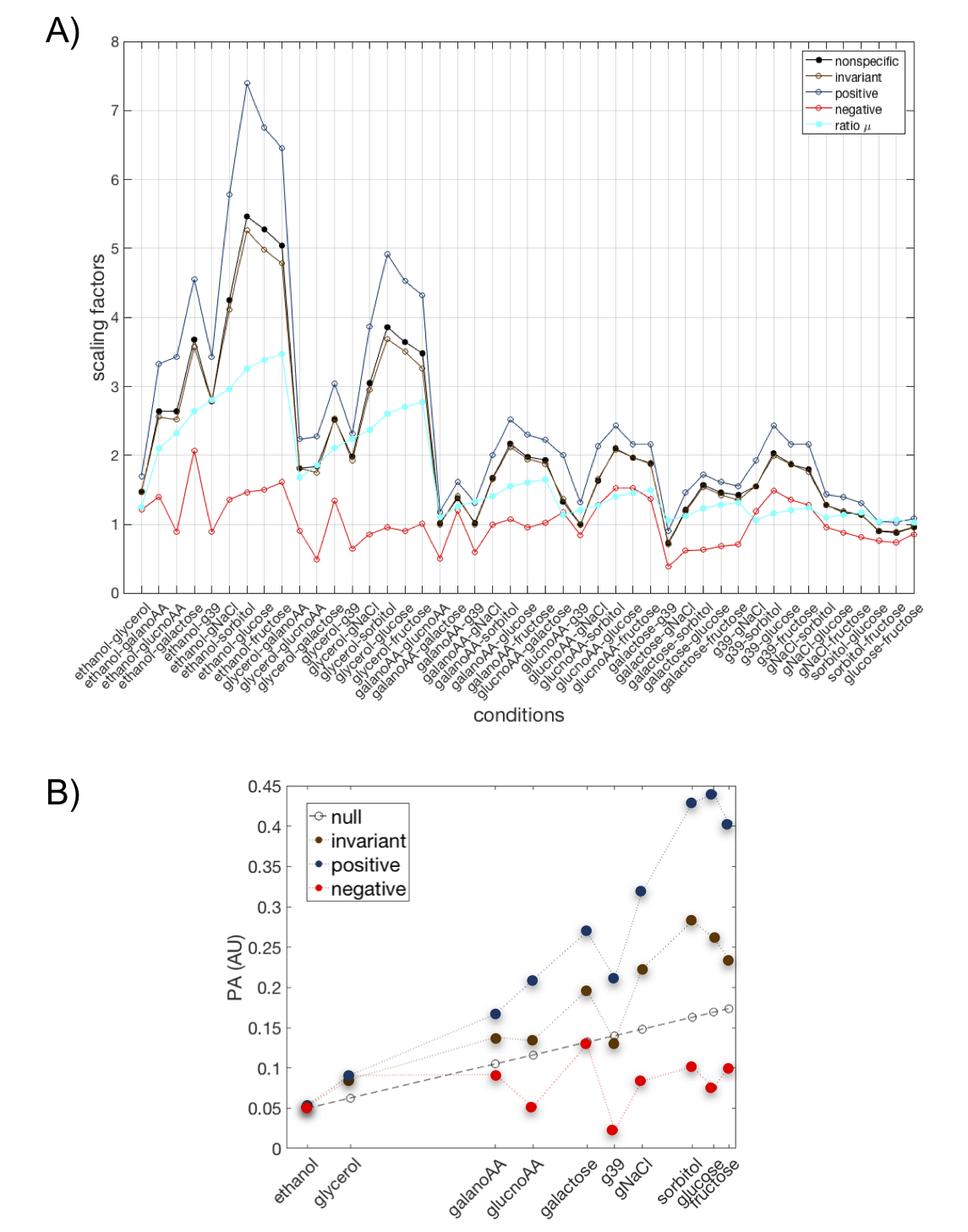

Supplement: S1 Fig — A) Genes that follow a single proportional scaling may serve a definite cellular function according to [11]. We find a single scaling that describes the change of promoter activity (PA) for different subsets of promoters according to the five-sector partition. The three classes within the nonspecific promoters (invariant, negative, positive) clearly show singular scaling. Shown also a null that corresponds to the ratio of growth rates between conditions (cyan curve). B) PA response of a typical invariant, positive and negative gene that corresponds to the mrs11, rps6A and atp5, respectively (conditions sorted by increasing growth rate; this is absolute PA not fractional PA). A null model of the dependence of PA with growth rate is given by the ratio of growth rates (empty circles). Gene categories within the global group clearly separate from the null. (TIF) [file pcbi.1007353.s001.tif]

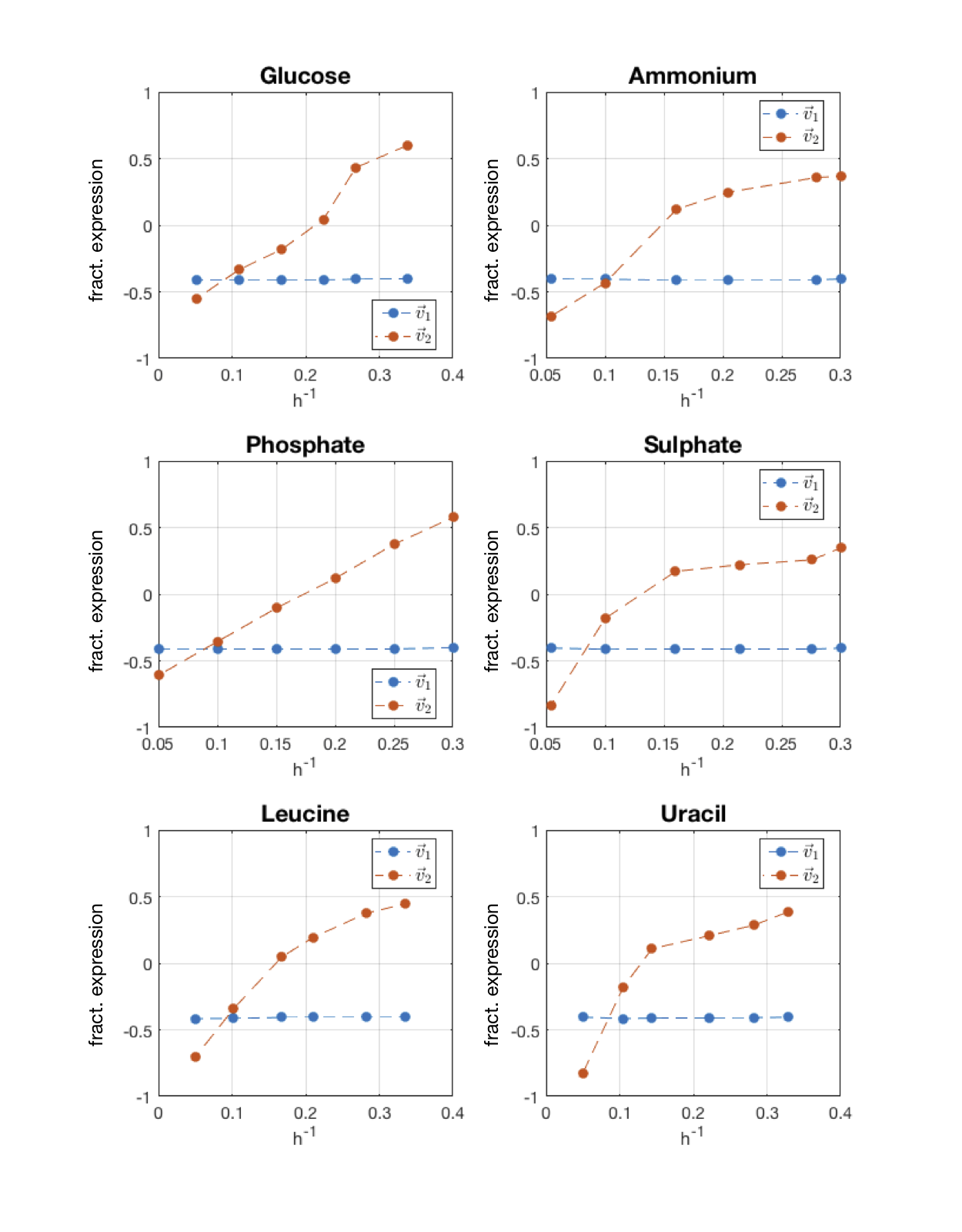

Supplement: S2 Fig — First and second SVD components exhibited an analogous trend in all conditions what underlines a core response. As a result, expression of each gene can be approximated by the linear combination of these two components on each nutrient. (TIF) [file pcbi.1007353.s002.tif]

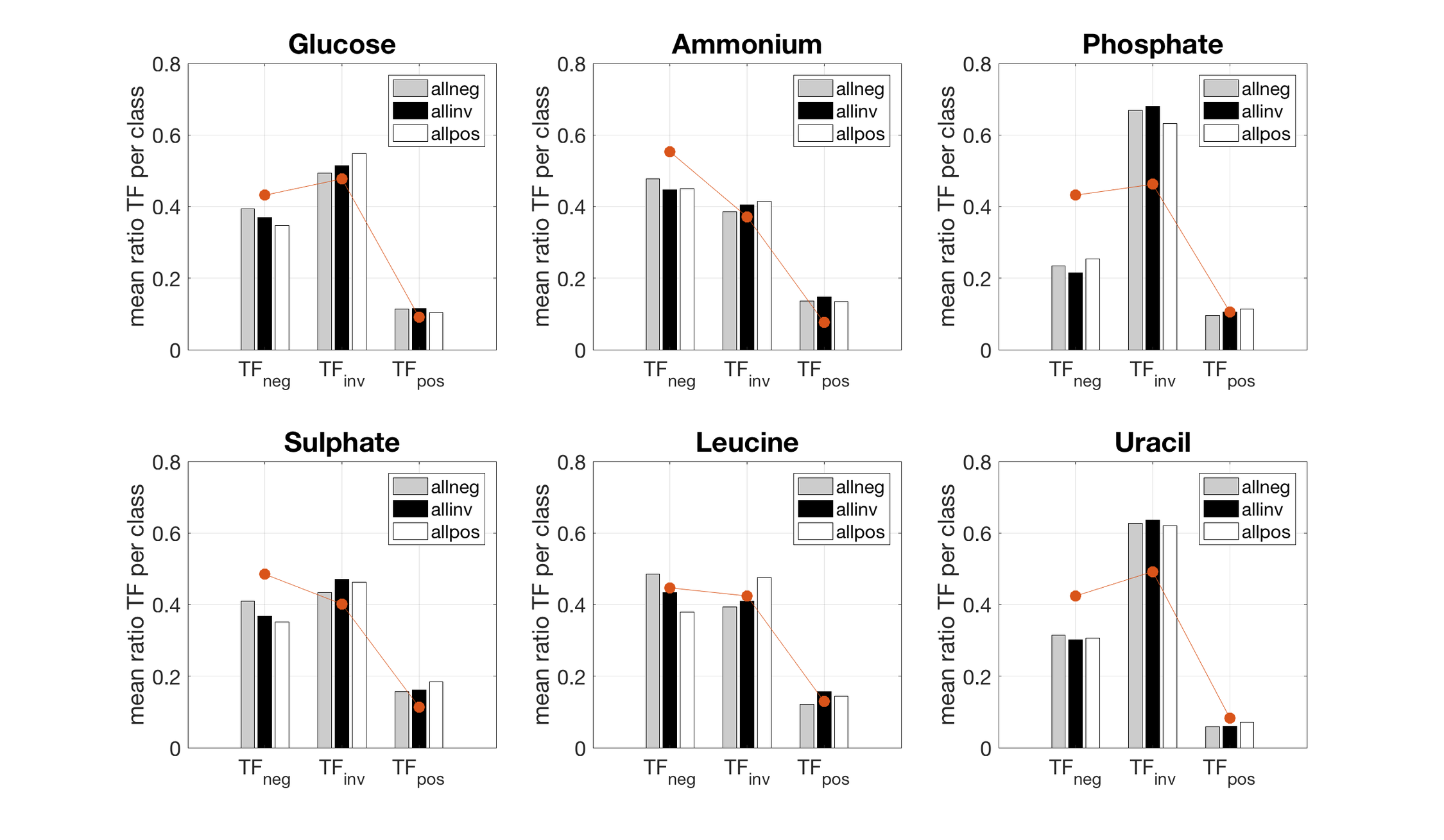

Supplement: S3 Fig — Fraction of TF class (negative/invariant/positive) acting on target genes divided also with respect to growth response (negative/invariant/positive; nonspecific and specific genes were included that we denoted as allneg, etc.). Mean values of each grouping are shown in bars, while the orange curves show the distribution of each class of TF on each condition. (TIF) [file pcbi.1007353.s003.tif]

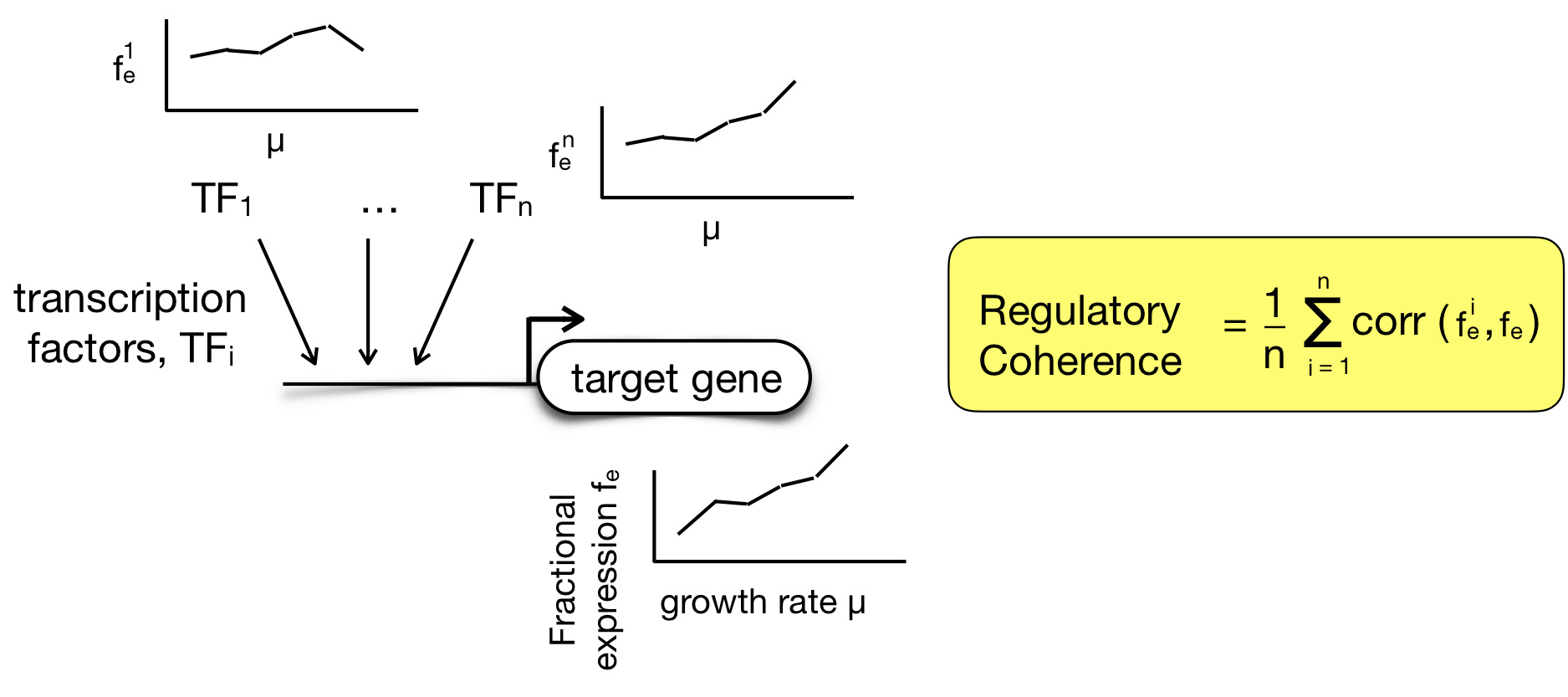

Supplement: S4 Fig — To estimate the active regulatory character of TFs, we measured the Pearson’s correlation of the response to growth rate between a particular target gene and all its cognate n TFs to then take the mean. This is the (mean) regulatory coherence in a given nutrient condition. (TIF) [file pcbi.1007353.s004.tif]

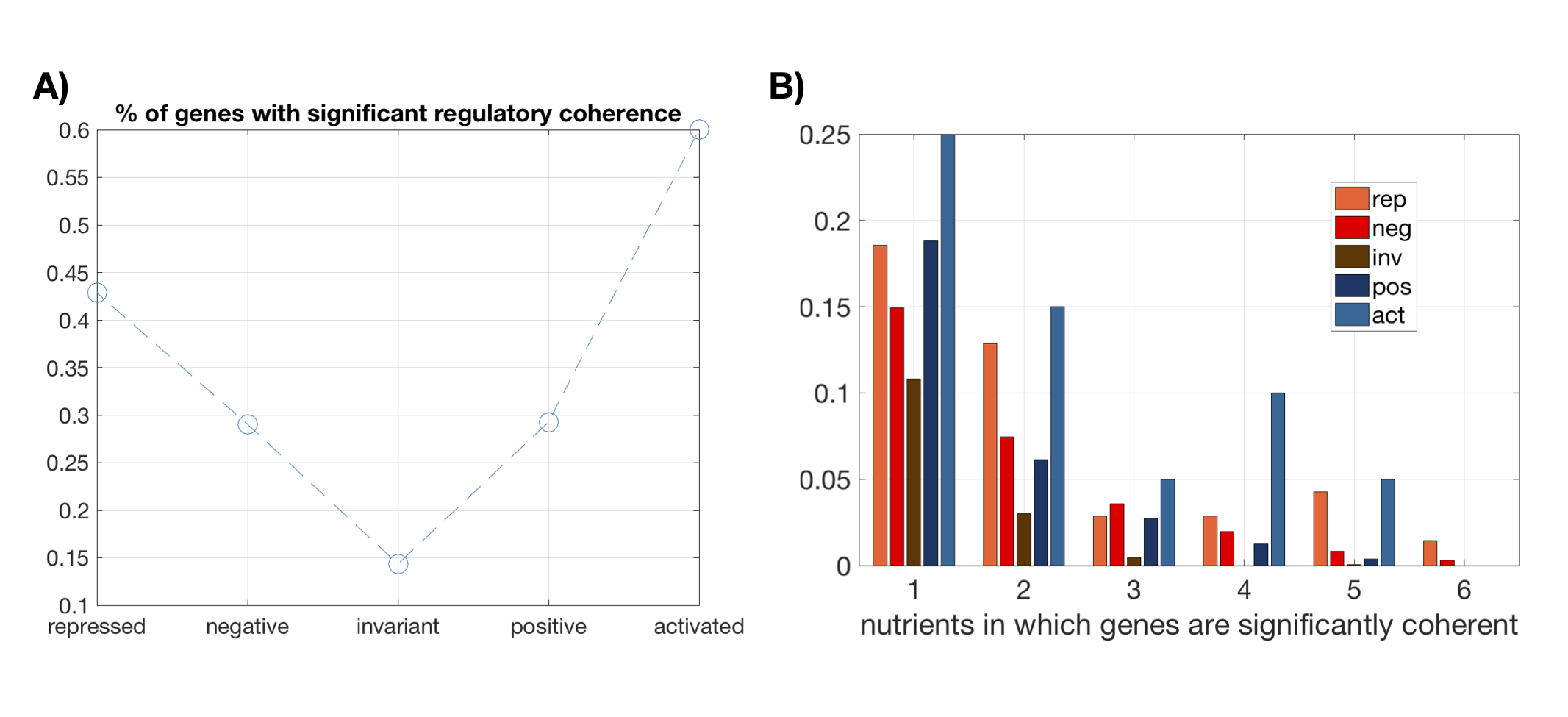

Supplement: S5 Fig — A) Percentage of genes within each class whose regulation is significantly coherent in at least one nutrient condition. Note that invariant genes show minimal coherence. B) Percentage of genes within each class that exhibits significant regulatory coherence in 1 to 6 different nutrient conditions. Specific genes (both repressed and activated) exhibit more cases of genes significantly coherent in more different conditions, while invariant genes show the opposite. See Methods, main text, for details. (TIF) [file pcbi.1007353.s005.tif]

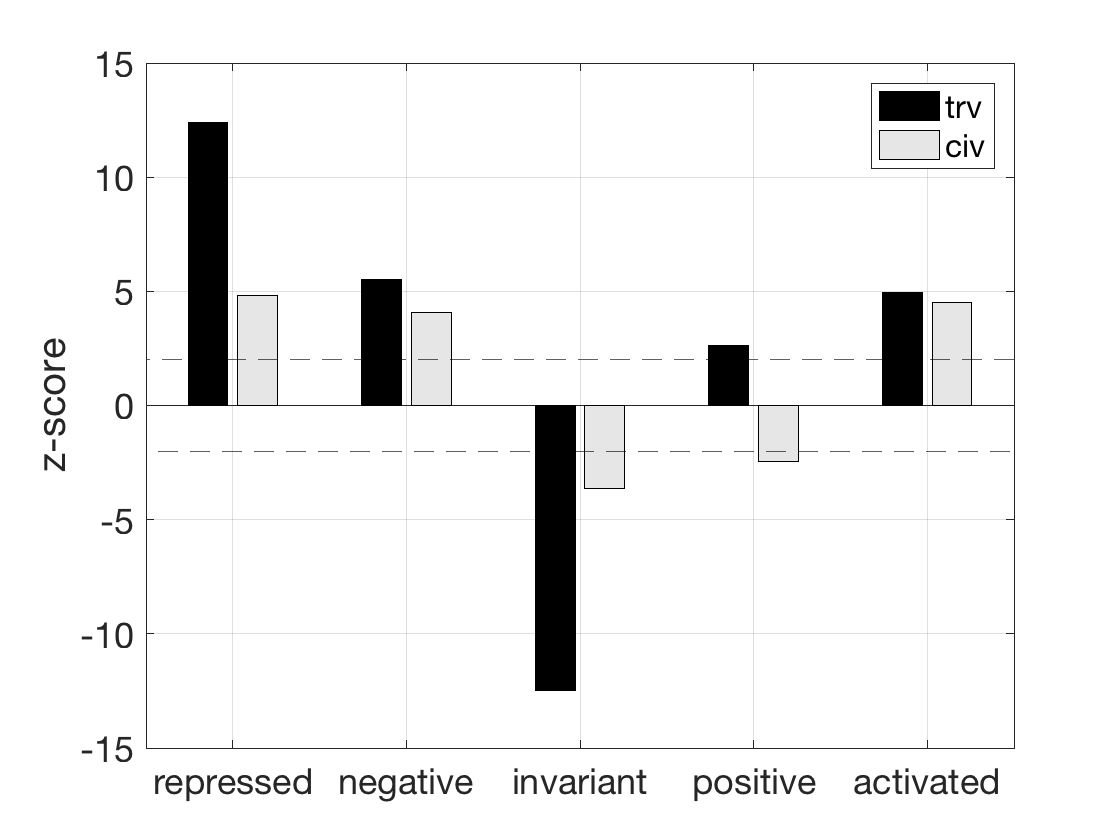

Supplement: S6 Fig — A cross between a standard laboratory yeast strain and a wild isolate allowed the computation of cis and trans effects on transcriptional variance [24]. For each partition, we quantified the mean of these measures and showed the associated z-score with respect to a null by randomization; dashed line indicates z-score = +/- 2. Positive genes show dominant effects associated with trans variability (trv and civ denote trans and cis variability, respectively). (TIF) [file pcbi.1007353.s006.tif]

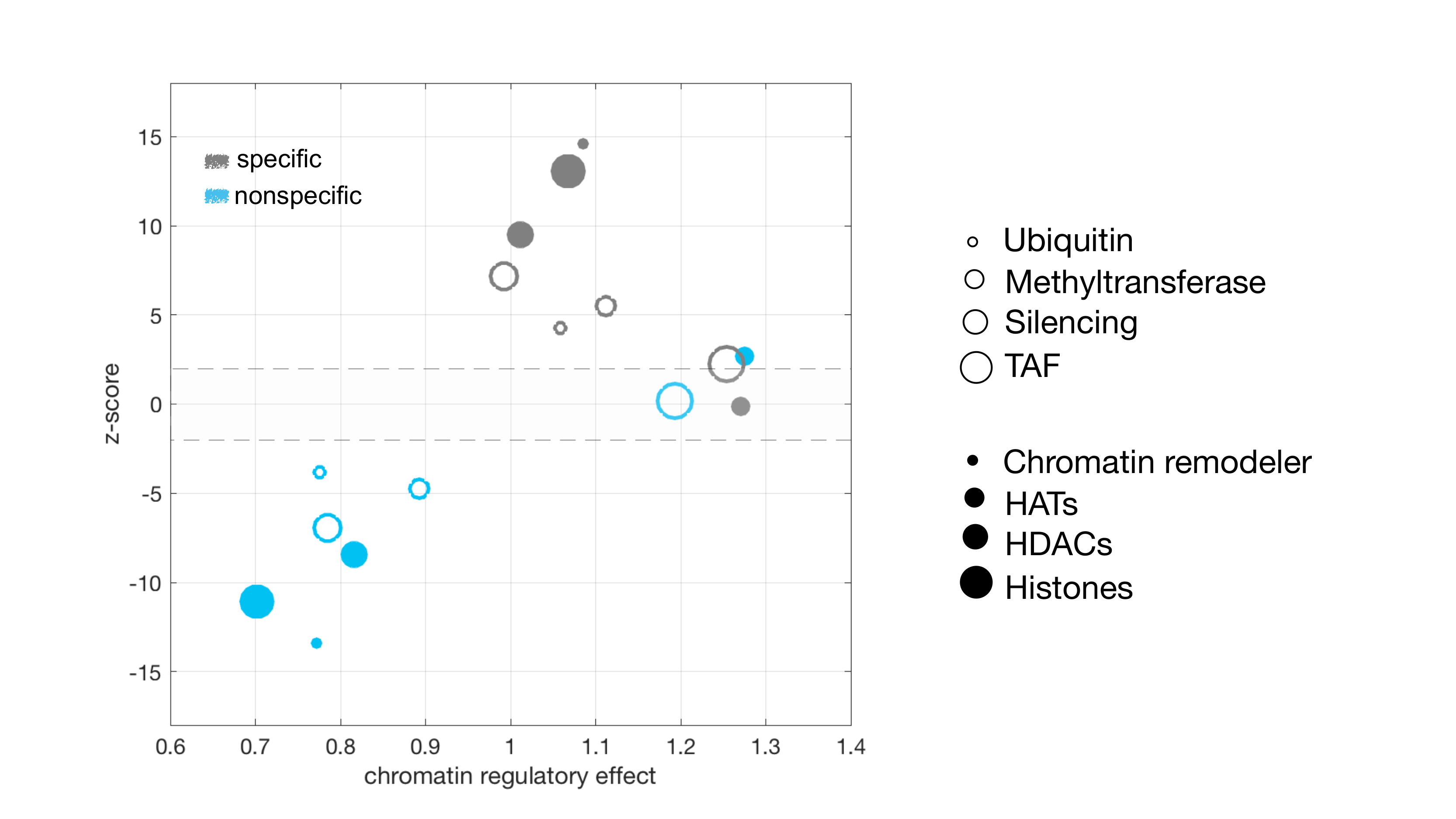

Supplement: S7 Fig — The chromatin regulatory effect (CRE; x axis) quantifies change in gene expression (absolute value) due to mutations in chromatin modifiers. CRE values larger than expected by a null (z-scores > 2, obtained by randomization) are observed for most modifiers on specific genes. Each circle type corresponds to a class of epigenetic modifier (TAF: TATA-binding protein related factors; HATs: histone acetyltransferases; HDACs: histone deacetylases); y axis denotes z-scores and dashed lines emphasizes z-scores within +/- 2 values. (TIF) [file pcbi.1007353.s007.tif]

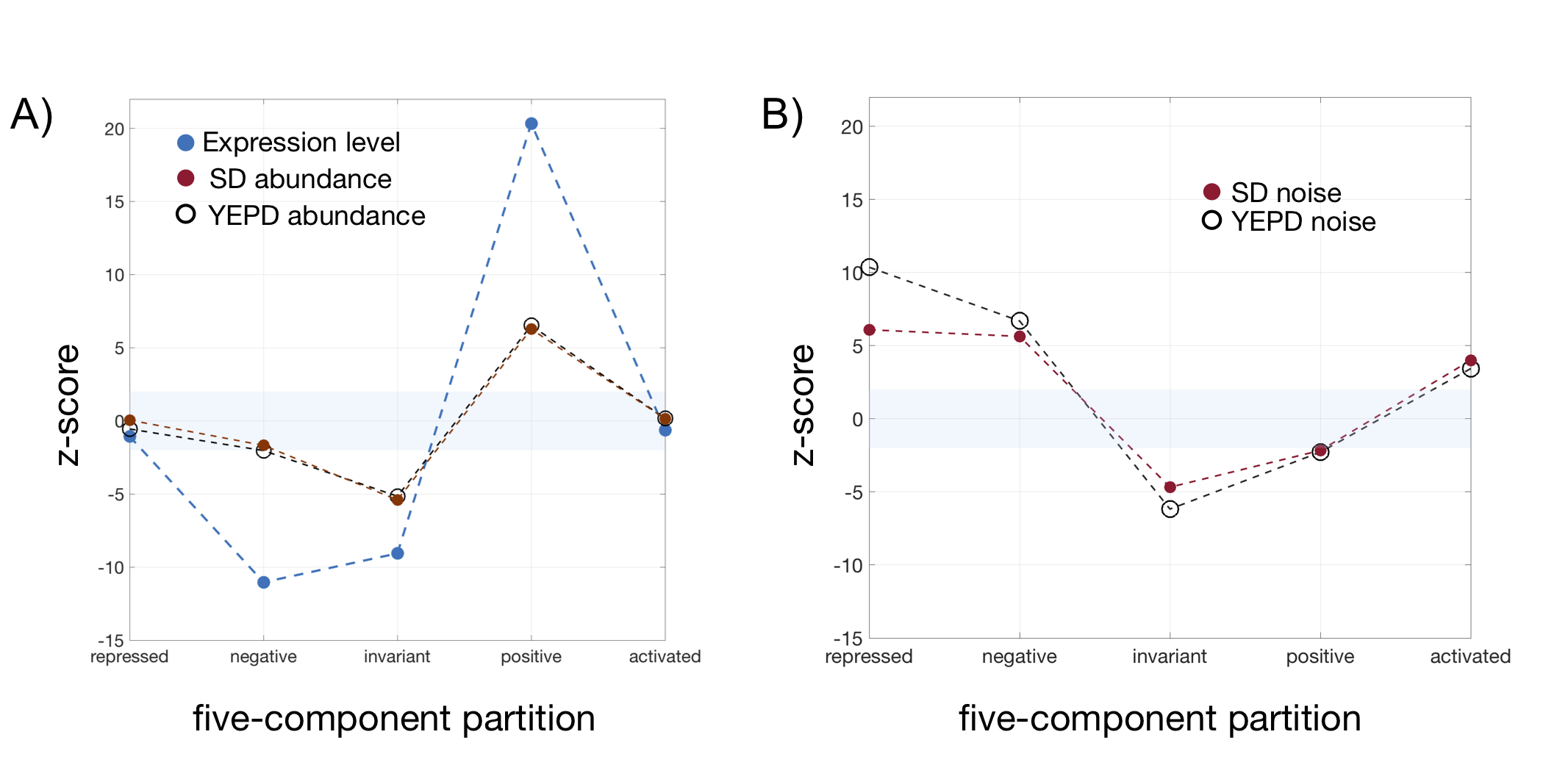

Supplement: S8 Fig — Mean expression and protein abundance (A) and protein noise (B) with respect to the five-sector partition as compared to a null in which classes were assigned randomly (10000 randomizations; y-axis is plotting the associated z-score, shading corresponds to z-score values within a range of -/+ 2; SD/YEPD denote poor/rich growing conditions). Nonspecific and positive genes showed high expression and low noise, a signal that was associated to the presence of fragile nucleosomes in the promoter and the action of general transcription factors [both enriched in nonspecific positive genes, see main text and [27] for details on data]. (TIF) [file pcbi.1007353.s008.tif]
